# Neuroanatomy of the Marine Jurassic Turtle *Plesiochelys etalloni* (Testudinata, Plesiochelyidae)

**DOI:** 10.1371/journal.pone.0069264

**Published:** 2013-07-02

**Authors:** Ariana Paulina Carabajal, Juliana Sterli, Johannes Müller, André Hilger

**Affiliations:** 1 Consejo Nacional de Investigaciones Científicas y Técnicas-Museo Carmen Funes, Neuquén, Argentina; 2 Consejo Nacional de Investigaciones Científicas y Técnicas-Museo Egidio Feruglio, Trelew, Chubut, Argentina; 3 Museum für Naturkunde Leibniz-Institut für Evolutions- und Biodiversitätsforschung an der Humboldt-Universität zu Berlin, Berlin, Germany; 4 Helmholtz-Zentrum für Materialien und Energie, Berlin, Germany; Ludwig-Maximilians-Universität München, Germany

## Abstract

Turtles are one of the least explored clades regarding endocranial anatomy with few available descriptions of the brain and inner ear of extant representatives. In addition, the paleoneurology of extinct turtles is poorly known and based on only a few natural cranial endocasts. The main goal of this study is to provide for the first time a detailed description of the neuroanatomy of an extinct turtle, the Late Jurassic *Plesiochelysetalloni* , including internal carotid circulation, cranial endocast and inner ear, based on the first digital 3D reconstruction using micro CT scans. The general shape of the cranial endocast of *P*. *etalloni* is tubular, with poorly marked cephalic and pontine flexures. Anteriorly, the olfactory bulbs are clearly differentiated suggesting larger bulbs than in any other described extinct or extant turtle, and indicating a higher capacity of olfaction in this taxon. The morphology of the inner ear of *P* *. etalloni* is comparable to that of extant turtles and resembles those of slow-moving terrestrial vertebrates, with markedly low, short and robust semicircular canals, and a reduced lagena. In *P* *. etalloni* the arterial pattern is similar to that found in extant cryptodires, where all the internal carotid branches are protected by bone. As the knowledge of paleoneurology in turtles is scarce and the application of modern techniques such as 3D reconstructions based on CT scans is almost unexplored in this clade, we hope this paper will trigger similar investigations of this type in other turtle taxa.

## Introduction

While the study of endocranial casts has a long history in paleontology (see 1 and references herein), it was only in the past 20 years that the development of non-invasive technologies such as computed tomography made it possible to reconstruct endocranial structures in extinct reptile forms on a broader scale (e.g. [2–11]). Most of these studies, which focused largely on dinosaurs, included not only brain morphology, but also inner ear structure, and cranial vasculature, allowing for detailed comparisons with extant representatives (e.g. [8,12–14]). Despite these methodological advances, however, knowledge on the morphology and evolution of the turtle brain still remains poor.

The turtle brain is housed in a tubular braincase, composed of the prootic, opisthotic, epipterygoid (present in basal and pancryptodiran turtles), basisphenoid, basioccipital, and exoccipital, and roofed by the supraoccipital, parietal and frontal. As in other reptiles, the brain is smaller than the cranial cavity and therefore the endocast only vaguely reflects its parts [15]. In extant turtles, the cranial endocast shows poorly marked cephalic and pontine flexures, and an extremely short olfactory tract (e.g. [16–19]). While the inner ear morphology has been described for a few extant turtles such as *Chelydraserpentina* , *Chrysemys* *scripta* , *Carettochelysinsculpta* and *Chelonoidis* *chilensis* [20,21], there is no description of the inner ear of any extinct form. However, the general morphology of the turtle inner ear consists of a labyrinth with short and dorsoventrally low semicircular canals and a short and conical lagena.

Natural or artificial cranial endocasts of extinct turtles are known for only a few taxa such as the chelonioid *Corsochelyshaliniches* from the Late Cretaceous of Alabama [16], the pelomedusoids *Bothremyscooki* and *B* *. barberi* from the Late Cretaceous of North America [17,18], and the Late Cretaceous baenid *Plesiobaenaantiqua* from Canada [19]. Studies of the endocasts of extant turtles are also scarce, including only the chelonioids *Chelonia* *mydas* [16,22], *Caretta* *caretta* [16], *Dermochelys* *coriaceae* *,* *Eretmochelysimbricata* , and *Lepidochelys* *kempii* [23], the chelydrid *Macrochelys* *temminckii* [24], and the pelomedusoid *Erymnochelys* *madagascariensis* [17].

The purpose of the present study is to provide a detailed description of the endocranial cavity and inner ear of the extinct turtle *Plesiochelysetalloni* using micro-computed tomography (CT), to compare it with other available information on endocranial casts of turtles. *Plesiochelysetalloni* is a pancryptodiran turtle [25] from the Late Jurassic (Upper Kimmeridgian–Lower Tithonian) lithographic limestones of Western Europe. Previous authors suggested plesiochelyids were living either in freshwater or coastal marine habitats [26–28], and oxygen isotope composition of plesiochelyid turtles recently supported the latter hypothesis given the taxon’s marine isotopic signature [29]. The new data from this study will serve as a basis for a better understanding of turtle brain anatomy and evolution.

## Materials and Methods

**Figure 1:**
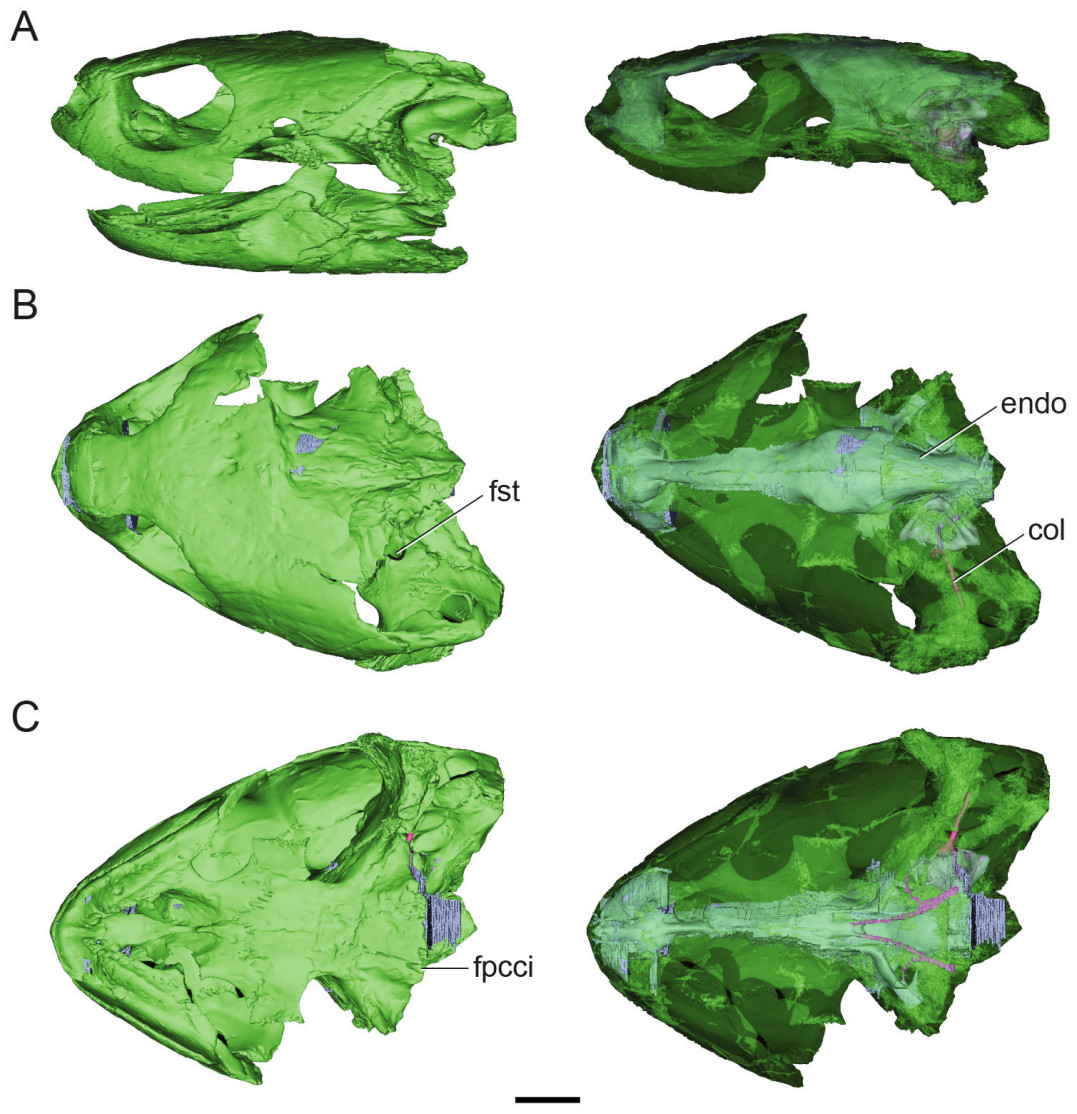
Volume-rendered CT-based reconstruction of the skull of the extinct turtle *Plesiochelysetalloni* (MH 435). In the images of the left side the bone is rendered opaque, whereas in the right side the bone is rendered semitransparent to show the endocranial cavity. The bone is shown in green, the cranial endocast and inner ear in light blue, the internal carotid arteries in pink, and the columella in fuchsia. Skull in left lateral (A) dorsal (B) and ventral (C) views. Abbreviations: col, columella auris; endo, cranial endocast; fpcci, foramen posterius canalis caroticus internus; fst, foramen staedio-temporale. Scale bar equals 10 mm.

*Plesiochelysetalloni* (MH 435) is represented by an almost complete skull (missing the occipital region) with the lower jaw [30,31] (Figure 1). This specimen was found in Glovelier (Canton de Berne) in Switzerland in 1950 and is housed in the collections of the Naturhistorisches Museum, in Basel, Switzerland. The species was described in detail by Gaffney [30,31] who based his description on the same specimen. Later, Sterli et al. [32] re-studied the basicranial region exploring the parabasisphenoid complex and the carotid circulation. To acquire the CT scans used in the present study, a loan of the specimen MH 435 was obtained from the Naturhistorisches Museum (Switzerland) to one of the authors (J. Müller) in March 2008. All necessary permits were obtained, and the study complied with all relevant regulations. For this study a high-resolution X-ray micro CT scan of the skull of MH 435 was performed at the Helmholtz-Zentrum für Materalien und Energie in Berlin, Germany, with 1000 projections over 360°, and exposure time of 0.6 s, a voltage of 100.0 kV, and current of 100 µA. Virtual three-dimensional inner ear and cranial endocasts were generated at the University of Alberta Paleovertebrate Laboratory (Alberta, Canada) using the software Materialise Mimics (14.0); and the resulting 3D models were then imported into the software Geomagic (10.0) to create 3D PDFs (see supplementary data). The final illustrations were generated using the software Illustrator (CS5). The endocast of *Plesiochelysetalloni* was compared with published turtle endocasts (e.g., [16,17,19]) and with an unpublished latex endocast of the terrestrial turtle *Chelonoidis* *chilensis* (Testudinidae, MPEF-AC 411). The CT data are archived at the Museo Carmen Funes (Plaza Huincul, Neuquén, Argentina) and at the Museum für Naturkunde Leibniz-Institut für Evolutions-und Biodiversitätsforschung an der Humboldt-Universität zu Berlin (Berlin, Germany).

## Description

### Cranial endocast

As in all extant and most extinct turtles, the anterior walls of the braincase such as ethmoidal elements, orbitosphenoids and laterosphenoids are not ossified, preventing the reconstruction of cranial nerves II to IV (cranial nerve I leaves an impression on the ventral aspect of the frontals). Endocranially, there is no floccular recess and the vestibular eminence is not ossified; consequently, the inner ear cavity is confluent with the endocranial cavity medially (*hiatus acusticus*, sensu [33]).

**Figure 2:**
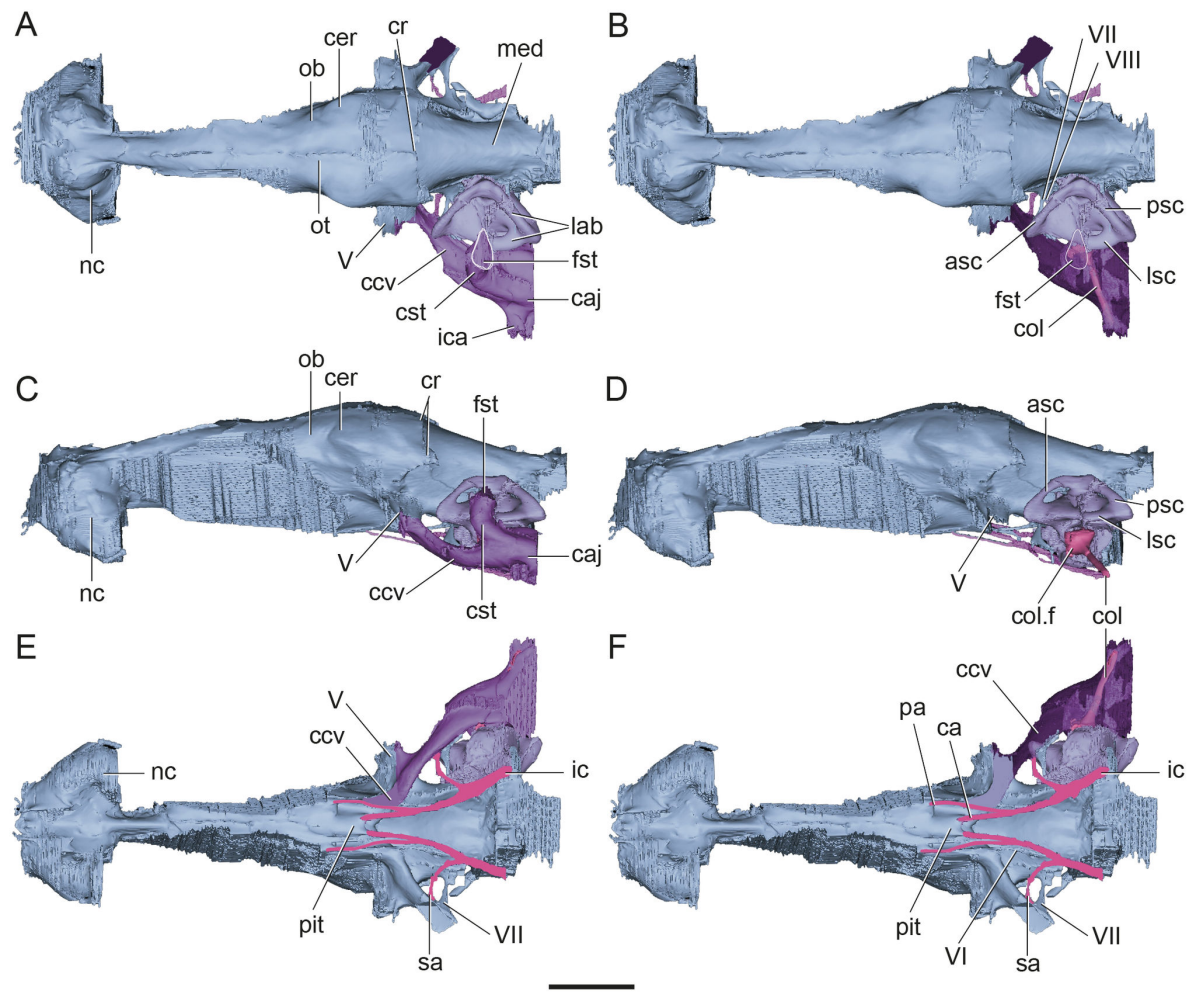
Surface-rendered CT-based reconstructions of the cranial endocast and endosseous labyrinth of *Plesiochelysetalloni* (MH 435). In dorsal (A–B), lateral (C–D), and ventral (E–F) views. The cavum acustico-jugulare is rendered opaque in (A, C, and E); to show the location of the columella it is rendered semitransparent in (B, F), and is absent in (D). The images show the cranial endocast in light blue, the inner ear in light violet, the cavum acustico-jugulare in purple, the columella in fuchsia, and internal carotids in pink. Abbreviations: asc, anterior semicircular canal; ca, cerebral artery; caj, cavum acustico-jugulare; ccv, canalis cavernosus; cer, cerebral hemisphere; col, columella auris; col. f, columellar foot; cr, cartilaginous ridge (= cartilaginous rider of [17,19]); cst, canalis stapedio-temporalis; fst, foramen stapedio-temporale; ic, internal carotid; ica, incisura columella auris; lab, endosseous labyrinth; lsc, lateral semicircular canal; med, medulla oblongata; nc, nasal cavity; ob, olfactory bulb; ot, olfactory tract; pa, palatine artery; pit, pituitary fossa; psc, posterior semicircular canal; sa, stapedial artery; V–VIII, cranial nerves. Scale bar equals 10 mm.

The cranial endocast of *P* *. etalloni* is almost complete, missing the posterior section of the medulla oblongata, together with the roots for cranial nerves IX to XII (Figure 1B, C). The shape of the endocast is generally similar to those of other extinct and extant turtles (e.g., [16,17,19]), being tubular and with poorly marked cephalic and pontine flexures. The preserved length of the endocast is 58.6 mm. The total estimated length would be 60.1 mm, based on the length of the endocasts of extant turtles (personal observations on an artificial endocast of *Chelonoidis* *chilensis* ). The hind-brain, midbrain and forebrain are more or less horizontally aligned as in the extinct *Corsochelyshaliniches* ( [15]: Figure 6B) and the extant *Dermochelys* *coriaceae* ( [23]: Figure 192); meaning the dorsal border of the medulla oblongata is in the same horizontal plane with most of the olfactory tract and nasal cavity when viewed from lateral (Figure 2). This differs from *Bothremys* ( [15]: Figure 7) and the extant sea turtles *Chelonia* *mydas* , *Lepidochelys* *kempii* , *Caretta* *caretta* , and *Eretmochelysimbricata* ( [23]: Figure 192) where the medulla is below the level of the olfactory tract.

#### Forebrain

The lateral and ventral walls of the olfactory tract cavity are not ossified. The olfactory tract is short, almost nonexistent, and 3.4 mm in width (maximum width). The impression of the olfactory bulb on the frontal, situated anteriorly to the tract, is shallow but clear enough to delimit its morphology. The bulbs are oval-shaped and slightly divergent from the midline (Figure 2A–D). The olfactory bulbs leave no impressions on the ventral aspect of the frontals in extant turtles (e.g. *Chelonioidis* *chilensis* personal observation) and for this reason the bulbs are not discernible in cranial endocasts. In the endocast of *P* *. etalloni* , the presence of olfactory bulbs indicates that those are relatively larger than in other studied turtles, suggesting that this taxon had a better olfaction. Cranial nerve I projects anteriorly in front of the olfactory bulbs in a trajectory that is slightly convex, and ends in the large nasal cavities. The nasal cavities are large and medially connected.

The cerebral hemispheres are easily discernible (Figure 2A). The maximum width of the endocast (13.3 mm) is at the level of the cerebral hemispheres, which are less laterally expanded than in *Bothremys* and *Corsochelyshaliniches* ( [15]: Figures 6A, 7B). On the ventral aspect of the endocast, the pituitary cast is small and dorsoventrally depressed (Figure 2E, F). The cerebral carotid arteries enter it posteriorly, through separated foramina (see below).

#### Midbrain

The optic lobes are not discernible in the endocast. The roots for cranial nerves II–IV cannot be reconstructed since the bones enclosing their foramina (orbitosphenoids) are not ossified in turtles [33].

#### Hindbrain

Dorsal to the cerebellum, there is a large triangular area marked by sharp ridges that are thickened laterally and connecting to the root of the trigeminal nerve, which are here interpreted as the cartilaginous ridge (= cartilaginous “rider” [17]) (Figure 2A–D). In other reptiles, this dorsal protuberance is related to the dorsal sagittal sinus, the dorsal head veins and the rostral middle cerebral vein (see 8. In *P*. *etalloni*, the cartilaginous ridge has marked lateral expansions that reach ventrally the root of CN V. The presence of this lateral ridge suggests that the dorsal head vein and the rostral middle cerebral vein drain through the same foramen as CN V. The dorsal longitudinal sinus is not well developed, although thick enough to hide the shape of the cerebral hemispheres. In the extant *Chelonia* *mydas* , Hopson [15] interpreted this area in the endocast as the cartilaginous anterior end of the supraoccipital (=cartilaginous “rider” of [17]).

**Figure 3:**
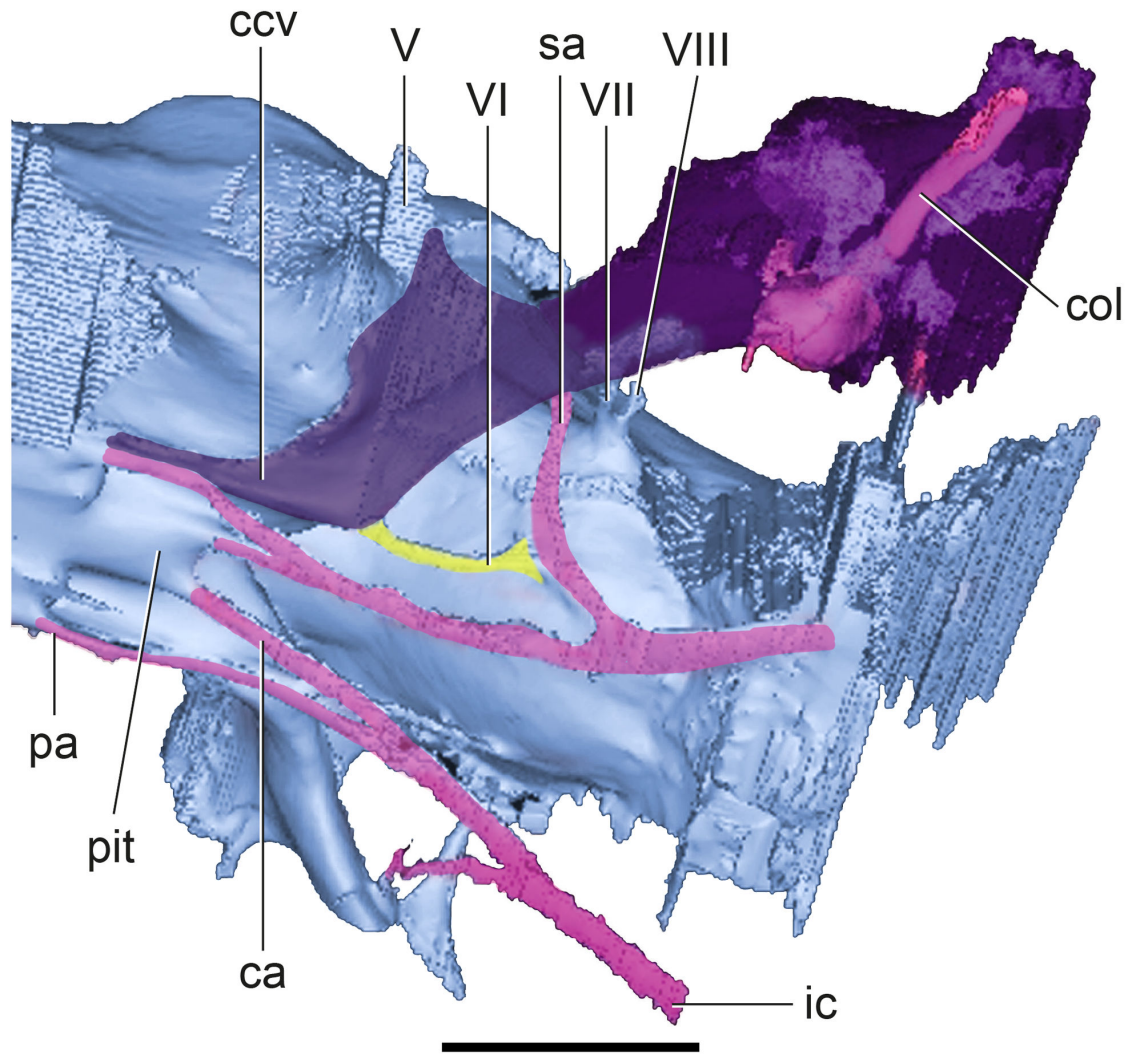
Detail of posterior region of the CT-based reconstructions of the cranial endocast of *Plesiochelysetalloni* (MH 435) in left anteroventral view. The figure shows the cranial endocast in light blue, the columella in fuchsia, the internal carotid arteries in pink, the CN VI in yellow, and the cavum acustico-jugulare and the canalis stapedio-temporalis (rendered semitransparent to show the *Columella* ) in purple. Abbreviations: ca, cerebral artery; ccv, canalis cavernosus; col, columella auris; ic, internal carotid; pa, palatine artery; pit, pituitary fossa; sa, stapedial artery; V–VIII, cranial nerves. Scale bar equals 10 mm.

Cranial nerve V leaves the endocranial cavity through a single large foramen (=foramen nervi trigemini [33]), enclosed mainly by the parietal with small contributions of the epipterygoid and the pterygoid [30,31]. The trigeminal root is large and posteroventrally in connection with the canalis cavernosus, which, at this level, is ventromedially projected (Figures 2A–C, 3).

The passage of CN VI has a very small diameter. In the endocast, CN VI exits at the base of the medulla and it passes through the basisphenoid in a dorsally convex trajectory to exit at the base of the processus clinoideus [33] close to the base of the most anteroventral section of the canalis cavernosus at the level of CN V (Figure 3). Both rami of CN VI pass almost parallel into anterior direction. The total length of the nerve is 5.5 mm.

**Figure 4:**
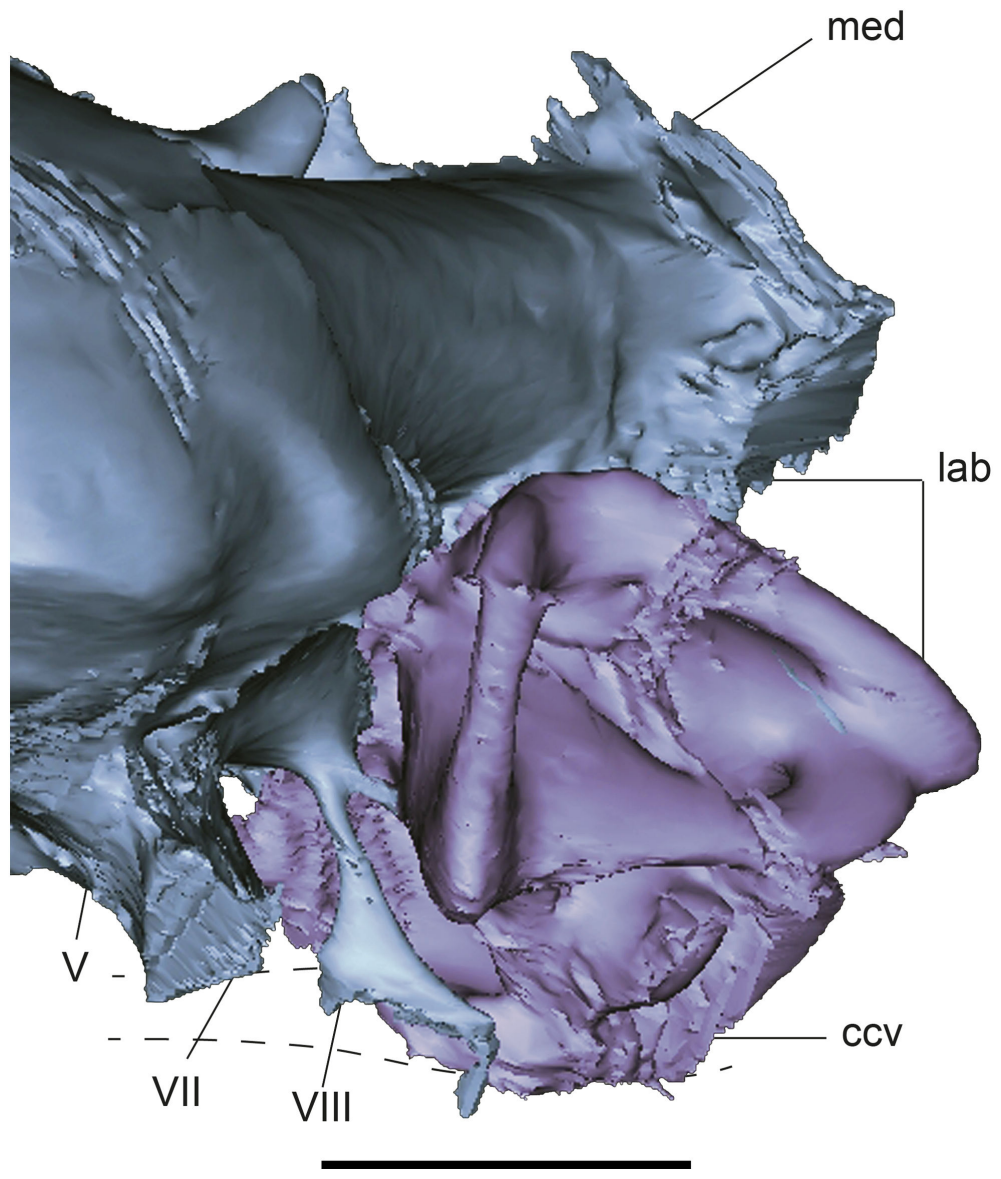
Detail of posterior region of the CT-based reconstructions of the cranial endocast and inner ear of *Plesiochelysetalloni* (MH 435) showing cranial nerves VII and VIII. The image shows the cranial endocast in light blue and the inner ear in light violet. Abbreviations: ccv, canalis cavernosus; lab, endosseous labyrinth; med, medulla oblongata; V–VIII, cranial nerves. Scale bar equals 10 mm.

Endocranially, cranial nerves VII and VIII are located posteroventrally to CN V (Figures 2B, 4). As in most turtles [33], they are housed in an oval, single recess (= fossa acustico-facialis [33]), and as revealed by the endocast, both nerves seem to share a single space, as product of the filling of the recess in the cast (Figure 3). CN VII leaves the recess anterior to CN VIII and has a larger diameter and a longer passage that passes laterally to enter the canalis cavernosus (Figure 2E). On the contrary, CN VIII has a smaller diameter and a shorter passage to enter the anterior ampulla of the inner ear. In turtles, usually two branches of CN VIII pierce the prootic, and sometimes a third branch enters the inner ear through the cartilaginous hiatus acusticus [33]. In the extant *Chelydraserpentina* , there are three branches of CN VIII, which are innervating the anterior ampulla, the macula sacculi and the posterior ampulla, respectively ( [34]: Figure 49). In *P* *. etalloni* , however, the preservation of a single foramen may be related to the lack of ossification of the vestibular eminence, although there is no evidence of a separated ganglion for other branches.

**Figure 5:**
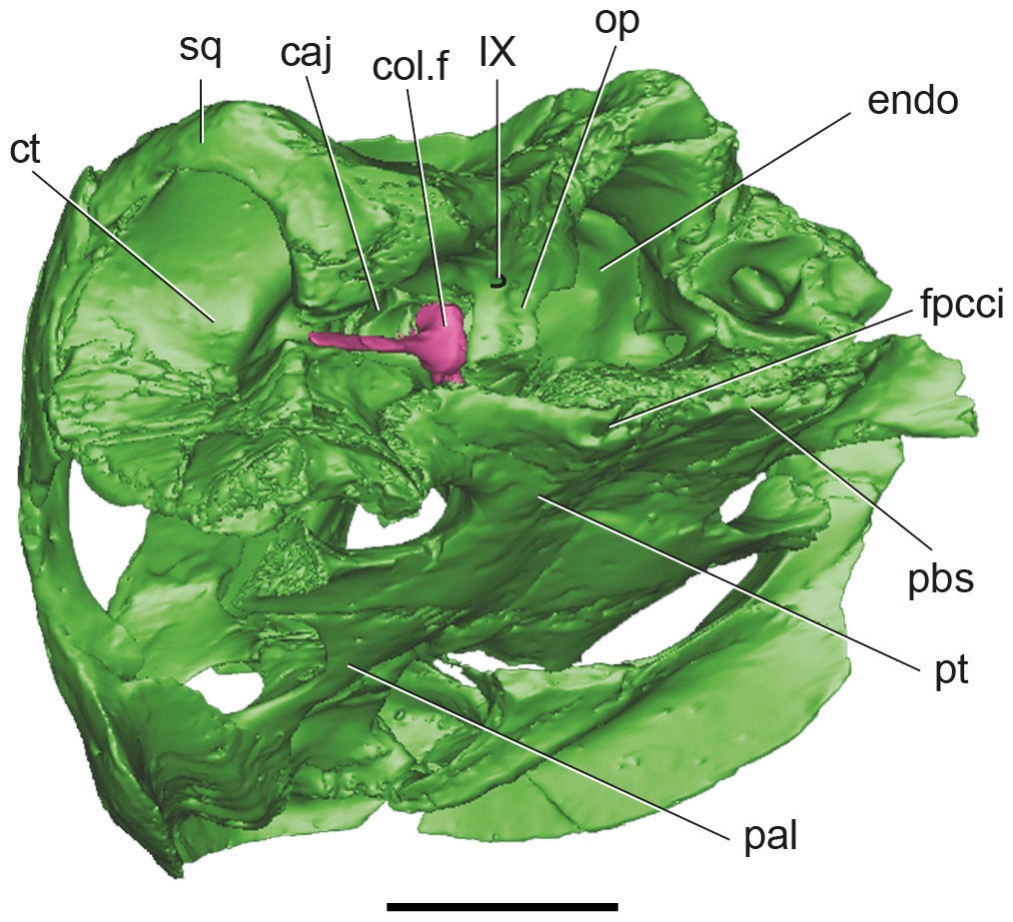
Digital reconstruction of the skull of *Plesiochelysetalloni* (MH 435) in lateroposteroventral view. Detail of the posterior part of the middle ear showing the foramen for cranial nerve IX. The image shows the bone in green and the columella in fuchsia. Abbreviations: cavum acustico-jugulare; col. f, columellar foot; ct, cavum tympani; endo, endocranial cavity; fpcci, foramen posterius canalis caroticus internus; op, opisthotic; pal, palatine; pbs, parabasisphenoid complex; pt, pterygoid; sq, squamosal; IX, cranial nerve. Scale bar equals 10 mm.

Only a short part of the medulla oblongata is preserved in *P* *. etalloni* , which is horizontally oriented (Figure 2C, D). As in all turtles, CN IX pierces the processus interfenestralis of the opisthotic (Figure 5). Due to the missing posterior part of the skull of *P* *. etalloni* , the trajectories of cranial nerves X to XII are not preserved.

### Inner ear

**Figure 6:**
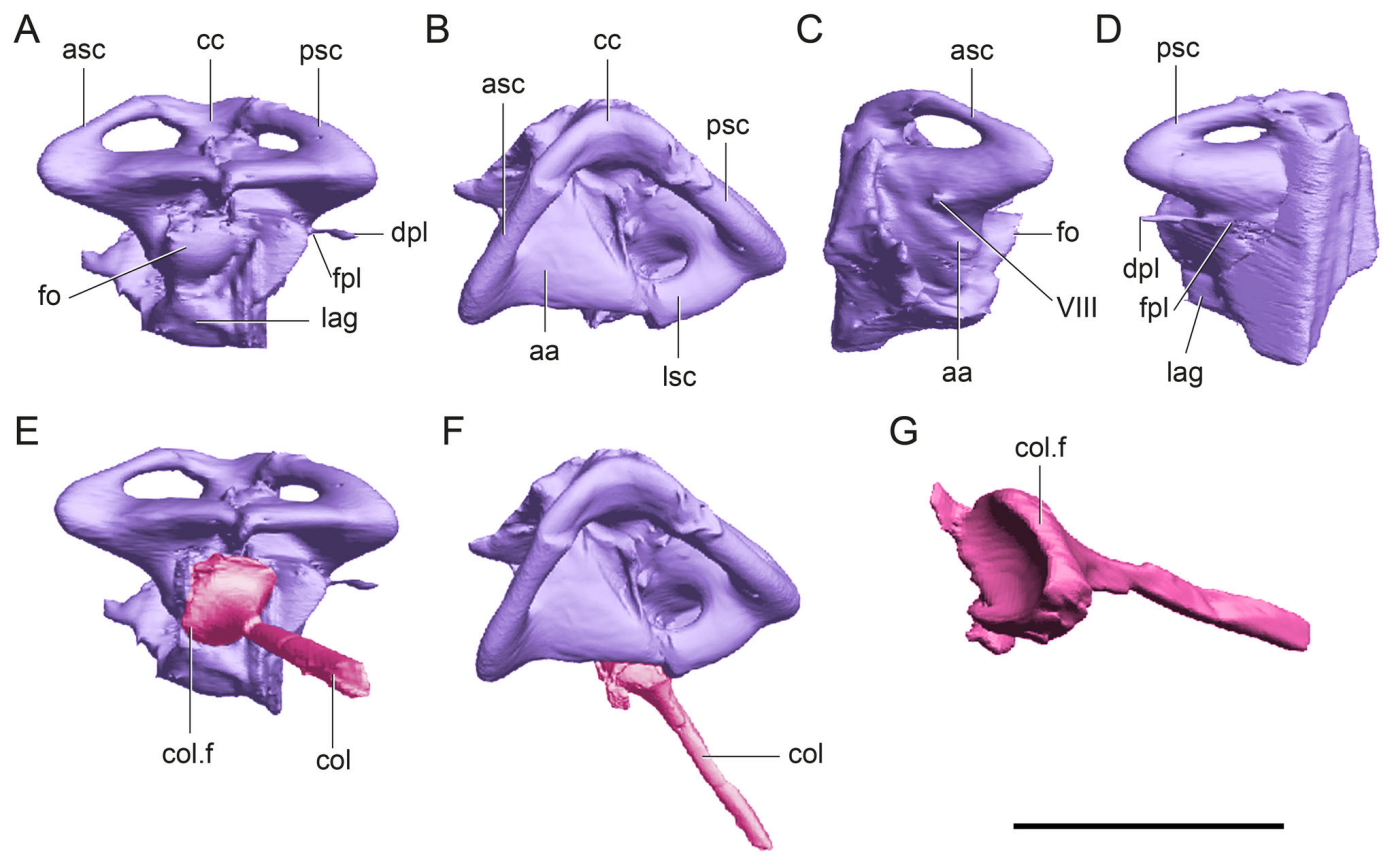
Digital reconstruction of the left inner ear of *Plesiochelys etalloni* (MH 435). In lateral (A, E), dorsal (B, F), anterior (C) and posterior (D) views. The image shows the inner ear in light violet and the columella in fuchsia. The columella is shown in anatomical position (E, F) and isolated in posteroventral view (G). Abbreviations: aa, anterior ampulla; asc, anterior semicircular canal; cc, common crus; col, columella auris; col. f, columellar foot; dpl, ductus perilymphaticus; fo, fenestra ovalis; fpl, fenestra perylimphatica; lag, lagena; lsc, lateral semicircular canal; psc, posterior semicircular canal; Scale bar equals 10 mm.

The complete left inner ear of *P* *. etalloni* was digitally reconstructed (Figure 6). The overall inner ear morphology corresponds to that described in extant turtles such as *Chelydraserpentina* ( [21]: Figure 1) and *Trachemys* *scripta* ( [35]: Figure 23-3), in which the lagena is globose and extremely short, and the semicircular canals are short, subequal in size and oval-shaped.

In *P* *. etalloni* , the anterior and posterior semicircular canals are markedly depressed dorsoventrally and the internal diameter is eye-shaped (Figure 6A, C, D). The diameter of the tube of the semicircular canals is as follows: 1.1 mm for the anterior semicircular canal (ASC), 1.2 mm for the posterior semicircular canal (PSC) and 1.7 mm for the lateral semicircular canal (LSC) (Figure 6B, F). The ASC is slightly larger than the PSC, whereas the LSC is the most robust and the smallest. The internal diameter of the ASC is 4.1 mm, while the PSC is 3.4 mm, and the LSC is 2.3 mm. The angle between ASC and the PSC is approximately 80° in dorsal view (Figure 6 B, F), which is slightly wider than the angle described for the extant turtle *Trachemys* *scripta* [2]. The anterior ampulla (=recessus labyrinthicus prooticus [33]) is well developed and larger than the posterior ampulla (=recessus labyrinthicus opisthoticus [33]). There is a small passage passing from the posterior ampulla to exit through a small foramen on the posterior part of the skull, the fenestra perilymphatica, opening between the cavum labyrinthicum and the recessus scalae tympani of the cavum acustico-jugulare formed by the opisthotic (Figure 6A, D). The periotic sac of the inner ear extends through this fenestra [33].

The fenestra ovalis is large and circular, occupying more than the 50% of the width of the vestibulum (Figure 6A). The diameter of the fenestra ovalis is 2.5 mm. The limit between the cavum labyrinthicum (inner ear) and the cavum acustico−jugulare (middle ear) is marked by the fenestra ovalis [33]. As in *Proganochelysquenstedti* , *Palaeochersistalampayensis* , and *Australochelys* *africanus* [36–38] and contrary to most extant turtles, the fenestra ovalis is completely surrounded by bone, anteriorly by the prootic and posteriorly by the opisthotic. In turtles, the elements forming the floor of the medial aspect of the inner ear are variable. In pancryptodires the floor of the inner ear is usually formed by the prootic and opisthotic, with occasional contributions from the basisphenoid, basioccipital, or pterygoid, while in many pleurodires, the floor of the cavum labyrinthicum is mainly cartilaginous with osseous contributions of the basisphenoid and quadrate [33]. In *P* *. etalloni* the floor of the cavum labyrinthicum is formed by bone. The basisphenoid forms the anteromedial quarter of the flooring of the inner ear, whereas the basioccipital and the pterygoid are closing the cavity posteromedially and ventrally, respectively (Figure 5).

As in other turtles, the lagena in *P* *. etalloni* is globose and markedly short (Figure 6A, D). The lagena houses the cochlear tube, which serves for sound perception [9]. Its length is directly related to the length of the sensory epithelium, the distance of which is used to estimate auditory capacity [9]. Recent studies on hearing showed that turtles have the lowest hearing frequency range within extant reptiles [21].

### Columella auris

The columella is almost completely preserved and in its natural position (Figure 2 B, D). It is nail-shaped, with a large and circular foot that is concave towards the fenestra ovalis. This foot is 2.5 mm in diameter. In *P* *. etalloni* , the shaft of the columella is a rod-like element (cylindrical) and 0.9 mm in diameter. The preserved length of the columella is 9.2 mm, and is only missing the extracolumella, which was probably cartilaginous, as in extant turtles [33].

### Canalis stapedio-temporalis and cavum acustico-jugulare

Regarding the middle ear cavity proper, the canalis stapedio-temporalis is posterodorsal and the canalis cavernosus of the cavum acustico-jugulare is lateral and anteroventral. The canalis stapedio-temporalis and the canalis cavernosus are confluent at the level of the inner ear (laterally to it), forming a single cavity located ventrolaterally to the hindbrain, as shown by the 3D reconstruction (Figure 2A–C, E, F).

The posterodorsal branch is named canalis stapedio-temporalis, and is a dorsally projecting passage that ends in the large foramen stapedio-temporalis on the posterior margin of the supratemporal fossa (Figures 1B, 2A, B). The canalis stapedio-temporalis and the foramen stapedio-temporale are formed by the prootic and quadrate. The arteria stapedialis passes through this canal [33], as probably did the hyomandibular branch of the nerve VII.

The cavum acustico-jugulare in turtles, is L-shaped in dorsal view (Figure 2A, B). The cavum acustico-jugulare is located between the cavum tympani (a funnel-shaped cavity formed by the quadrate in turtles) and the cavum labyrinthicum [33]. The elongated anteroventral part of the cavum acustico-jugulare corresponds to the canalis cavernosus (Figure 2A, C, F) and represents the cranio-quadrate space of basal tetrapods [33]. The posteromedial section of the cavum-acustico jugulare is the recessus scalae-tympani [33]. The recessus scalae-tympani is separated from the remaining parts of the cavum acustico-jugulare by the processus interfenestralis of opisthotic. The columella auris is housed by the cavum tympani (laterally) and in part by the cavum acustico-jugulare (Figures 2B, F, 5).

In *Plesiochelysetalloni* as in other turtles, the canalis cavernosus is the anteroventral branch and the largest (Figure 2A–C). It is arc-shaped and passes anteriorly to reach the level of the trigeminal nerve. The canalis cavernosus in *P*. *etalloni* passes then anteroventrally and medially and merges with its counterpart anteriorly to the pituitary fossa on the ventral aspect of the cranial endocast (Figure 2E, F). Cranial nerves V, VII, and the stapedial artery (see below) enter the lateral section of the canalis cavernosus, whereas CN VI and the palatine artery enter the ventral section of the canal (Figure 3), and the vena capitis lateralis passes inside the canal (cranio-quadrate space of other vertebrates sensu [33]).

### Carotid circulation

The internal carotid artery enters the skull at the posterior end of the pterygoid [32,39] (Figures 1C, 5). It passes anteriorly for a short length before it bifurcates into two branches (Figure 3). The medial branch continues anteriorly, whereas the lateral (arteria stapedialis) curves towards the canalis cavernosus. The arteria stapedialis enters the ventral aspect of the cavum acustico-jugulare. We assume that it passes posteriorly turning dorsally and entering the aditus canalis stapedio-temporalis, running along the canalis stapedio-temporalis to exit through the stapedio-temporalis foramen (Figures 2E, F, 3). The medial branch of the internal carotid continues anteriorly for a short distance and bifurcates again into lateral and medial branches. The medial branch (arteria *cerebralis*) enters the pituitary fossa posteriorly (Figures 2E, F, 3). The lateral branch (arteria *palatina*) passes anterodorsally and enters the canalis cavernosus anteriorly to the pituitary fossa (Figures 2E, F, 3). This arterial pattern, where all the internal carotid branches are enclosed by bone, corresponds to pattern I as recognized by Sterli and de la Fuente [40].

## Discussion and Conclusions

The scarce knowledge of endocranial casts in turtles prevents a more detailed comparison of the endocranial cast of *P* *. etalloni* with that of other turtles. We hope this work will be the starting point for more descriptions of endocranial reconstructions using extinct and extant turtles.

**Figure 7:**
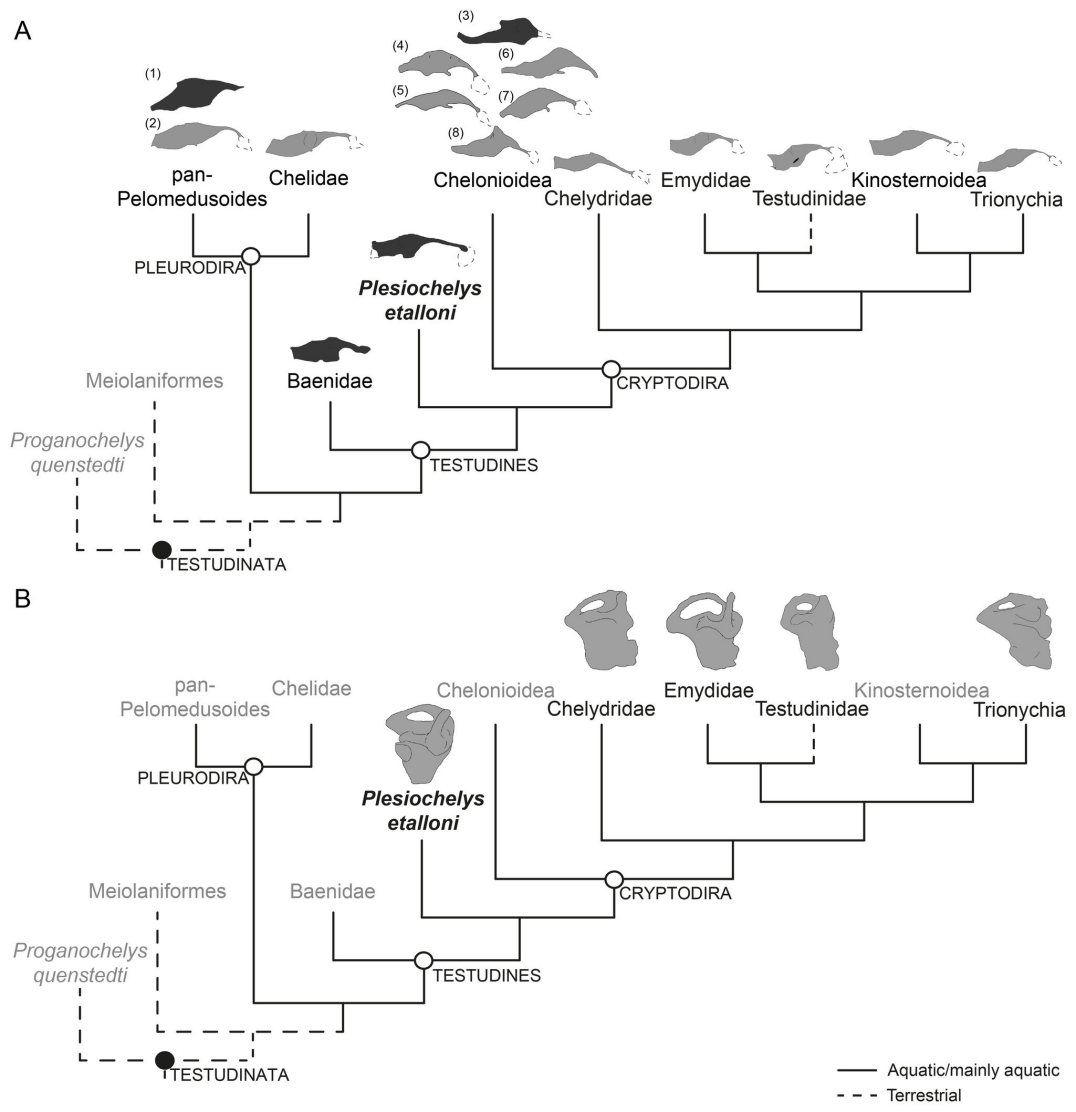
Brain endocasts and left endosseous inner ears of turtles available in the literature displayed in a simplified cladogram (based on Joyce [25]). The black circle indicates a synapomorphy based clade definition while the white circle indicates a crown-group definition following Joyce et al. [54]. A, Brain endocasts comparison based on line drawings of extinct and extant turtles in right lateral view represented by (left to right) (1) *Bothremys cooki* (redrawn from [17]), (2) *Podocnemis unifilis* (after [33]), Chelidae *Emydura macquarii* (after [33]), Baenidae *Plesiobaena antiqua* (redrawn from [19]), *Plesiochelys etalloni* (this study), (3) *Corsochelys haliniches* (redrawn from [16]), (4) *Chelonia mydas*, (5) *Eretmochelys imbricata*, (6) *Caretta caretta*, (7) *Lepidochelys kempi*, (8) *Dermochelys coriacea* (after [23]), Chelydridae *Macrochelys temminckii* (after [33]), Emydidae *Pseudemys concinna* (after [33]), Testudinidae *Chelonoidis chilensis* (this study), Kinosternidae *Sternotherus odoratus* (after [33]), and Trionychia *Apalone ferox* (after [33]). Dashed line indicates the shape of the nasal cavity. Dotted line in *Plesiochelys etalloni* indicates reconstructed area of the endocast. Dark gray, extinct species. Light gray, extant species. Not to scale. B, Inner ear endocasts were redrawn from the following sources: *Plesiochelys etalloni* from this study and Chelydridae, Emydidae, Testudinidae, and Trionychia from Thewissen and Nummela [20] (Fig. 15.11 K-N). Chelydridae is represented by *Macrochelys temminckii*; Emydidae is represented by *Trachemys scripta*; Testudinidae is represented by *Chelonoidis nigra*; Trionychia is represented by *Carettochelys insculpta*. *Proganochelys quenstedti*, Meiolaniformes, and *Plesiochelys etalloni* are extinct taxa. Not to scale.

In general aspects, the shape of the brain of *P*. *etalloni* is similar to that of *Dermochelys* *coriaceae* . This marine species has the most horizontally developed brains, closely resembling that of *P*. *etalloni*. Other marine species such as *Caretta* *caretta* , *Eretmochelysimbricata* , *Chelonia* *mydas* and *Lepidochelys* *kempi* have brains with well-marked cephalic flexures and nasal cavities that are long and ventrally convex [23] although a cranial endocast would probably not reflect this morphology. As observed in skull dissections on extant taxa [23] and as described for archosaurian reptiles, the brain parts are obscured by the overlying dural venous sinuses and the dura itself [8]. In this sense, the endocranial cavity does not reflect well the morphology of the brain and other soft tissues. For example, the pineal gland is well developed in most turtles, but only in *D* *. coriaceae* [23] is it correlated with a dorsal projection of the endocranial cavity (identified as the cartilaginous “rider” by Gaffney and Zangerl [17] (Figure 7A). In *P* *. etalloni* the cartilaginous rider is one of the most striking features on the dorsal surface of the endocast. This structure could indicate the location of the pineal organ, but also could be a part of the dorsal longitudinal sinus. Among extinct turtle species, the largest known cartilaginous ridge is observed in *Corsochelyshaliniches* and *Brothremyscooki* (Figure 7A), whereas it is less prominent in *P* *. etalloni* and *Plesiobaenaantiqua* .

Regarding the olfactory region, in extant turtles the small olfactory bulbs do not leave impressions on the frontals and cannot be clearly identified on the artificial endocasts [41]. However, the olfactory bulbs are clearly differentiated in the endocast of *P* *. etalloni* , suggesting larger bulbs than in any other described extinct or extant turtle, and indicating a relatively higher capacity of olfaction in this taxon.

In vertebrates it has been proven that the endosseous labyrinth closely follows the path and shape of the membranous semicircular ducts (see 42 and references cited there). Therefore, interpretations on hearing and balance in extinct taxa based on extant species seem to be adequate at this level. As in other vertebrates, the vestibular system is involved in the coordination of movement, gaze control, and balance, detecting head movement (head rotation) in space and maintaining visual and postural stability. For a given angular acceleration, the maximum achieved bending depends on the sensitivity of the semicircular canal (which in turns depends on the length of the canal, on the area enclosed by the canal in its maximal response plane, and on the cross-section of the semicircular duct and the ampulla) (see 42 and references cited there). In *P* *. etalloni* the labyrinth is dorsoventrally depressed and the diameters of the tubes are ASC<PSC<LSC (e.g. the internal diameter of the LSC is one and a half smaller than the ASC). Also, the fenestra ovalis seems to be more dorsally located (closer to the LSC) than in other turtles. The ASC is dorsoventrally depressed and markedly developed anteroposteriorly forming an angled margin with the anterior ampulla. This disposition is similar to the one observed in *Carettochelysinsculpta* and *Chelonoidis* *chilensis* (this study). The anterior ampulla is well developed. It housed the utriculus, being the section of the vestibulum that senses motion in the horizontal plane (e.g., forward, backward, and left–right movements).

The morphology of the inner ear of *P* *. etalloni* resembles those of other slow-moving terrestrial vertebrates such as the tuatara and the non-arboreal chamaeleon *Brookesia*, in which this morphology is optimized to detect weak accelerations, probably important to ensure postural stability [43]. Recent studies showed that in most turtle species, the turtle ear is less sensitive to airborne sound than those of other tympanate tetrapods [44]. However, aquatic turtles have better hearing than terrestrial turtles (i.e. the range of sound perception is higher) since the ear responds to much lower sound energy in water, and therefore being more efficient. Recent studies also showed that the turtle ear is adapted for underwater hearing, explaining both the poor sensitivity to airborne sound and the turtle middle ear structure [21,44]. For the turtle *Trachemys* *scripta* , the underwater audiograms had the same shape as audiograms in air, with best frequency of 400–500 Hz [44]. In terms of environmental adaptation the inner ear morphology in turtles is yet unclear. For Lenhardt et al. [45] the auditory process in turtles is even more complex. They suggested that the sound process in turtles involves the traditional air conduction, but also bone conduction. The sound captured by bone conduction happens through the vibration of skull and/or shell bones ( [45]), while air conduction occurs through the tympanum. Manley and Kraus [46] stated that hearing limits are much less specific to systematic groups than previously thought, depending less on phylogenetic position than on species-specific physical (size) and ecological (temperature regime, lifestyle) constraints. As stated by Christensen-Dalsgaard et al. [44] the behavioral significance of hearing in turtles still remains to be discovered, but navigation, prey detection and predator avoidance are probably important in this group, as in many other aquatic vertebrates. With reference to studies suggesting that the inner ear system of aquatic turtles is elongated anteroposteriorly relative to terrestrial turtles [20], our comparison of the endosseous labyrinth of *P* *. etalloni* indicates that the inner ear morphology is not useful to identify the taxon’s ancient habitat (Figure 7B). However, the sample of known inner ears of extinct turtles is so small that new descriptions are needed to understand the implications of its morphology (Figure 7B).

Another interesting structure clearly observed in the 3D reconstruction is the carotid circulation pattern. The different patterns of carotid circulation have long been used for turtle systematics (e.g., [25,39,40,47,48]). In recent years many new extinct turtles have been discovered and new studies have been made, highlighting that the complexity of the carotid circulation in turtles is greater than previously thought (e.g., [40,49–53]). The 3D endocranial reconstruction of *P* *. etalloni* reinforces the previous descriptions of its carotid circulation [30–32]. In *P* *. etalloni* the carotid circulation is similar to that found in extant cryptodires (pattern I of [40]). In these turtles the carotid artery enters the skull through the foramen posterius canalis caroticus internus located in the posterior part of the pterygoid. The entire path of the carotid artery, and its bifurcation into cerebral and palatine branches, is surrounded by bone.

## Supporting Information

File S1 3D PDF (PDF)
