# Correction: Neuroanatomy of the Marine Jurassic Turtle *Plesiochelys etalloni* (Testudinata, Plesiochelyidae)

**DOI:** 10.1371/journal.pone.0212241

**Published:** 2019-02-07

**Authors:** Ariana Paulina-Carabajal, Juliana Sterli, Johannes Müller, André Hilger

The first author's name is spelled incorrectly in the citation. The correct name is: Ariana Paulina-Carabajal. The correct citation is: Paulina-Carabajal A, Sterli J, Müller J, Hilger A (2013) Neuroanatomy of the Marine Jurassic Turtle Plesiochelys etalloni (Testudinata, Plesiochelyidae). PLoS ONE 8(7): e69264. https://doi.org/10.1371/journal.pone.0069264
