# Supplementary figures and images for: Neuroanatomy of the Marine Jurassic Turtle Plesiochelys etalloni (Testudinata, Plesiochelyidae)
Source: PLoS One. 2013 Jul 2;8(7):e69264. doi: 10.1371/journal.pone.0069264 (PMC3699497; doi:10.1371/journal.pone.0069264)

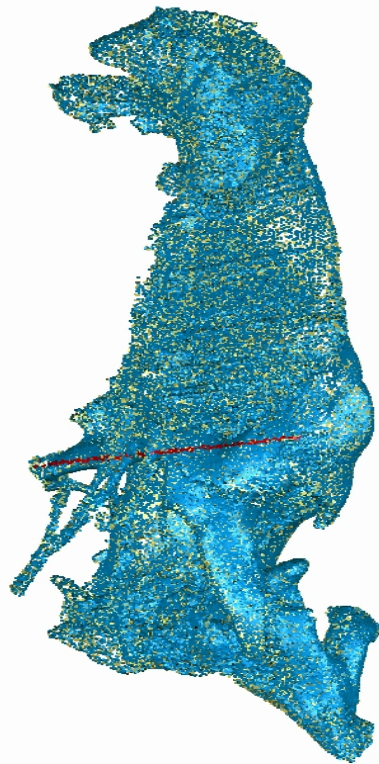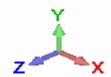

Click on the image to activate the 3D Model.

Supplement: File S1 — (PDF) [file pone.0069264.s001.pdf]
